# Comparison of the efficacy and safety of two starting dosages of prednisolone in early active rheumatoid arthritis (CORRA): study protocol for a randomized controlled trial

**DOI:** 10.1186/1745-6215-15-344

**Published:** 2014-09-02

**Authors:** Ulrike S Trampisch, Dietmar Krause, Hans J Trampisch, Renate Klaassen-Mielke, Xenofon Baraliakos, Jürgen Braun

**Affiliations:** Department of Medical Informatics, Biometry & Epidemiology, Ruhr-Universität Bochum, Universitätsstraße 150, D-44801 Bochum, Germany; Faculty of Health, School of Medicine, Institute of General Practice and Family Medicine, Witten/Herdecke University, Alfred-Herrhausen-Str. 50, D-58448 Witten, Germany; Rheumazentrum Ruhrgebiet, Claudiusstr. 45, D- 44649 Herne, Germany

**Keywords:** Rheumatoid arthritis, Glucocorticoids, Bridging therapy, Safety, Radiographic joint damage, Osteoporosis

## Abstract

**Background:**

Although glucocorticoids are widely used in the treatment of patients with early rheumatoid arthritis, the best dosage of glucocorticoids with regards to efficacy and safety is not known.

The aim of the study ‘Comparison of the efficacy and safety of two starting dosages of prednisolone in early active rheumatoid arthritis’ (CORRA) is to compare two standard glucocorticoid starting dosages and the non-use of glucocorticoids in the treatment of patients with early active rheumatoid arthritis on the background of the established ‘anchor’ therapy with methotrexate.

**Methods/design:**

CORRA is an investigator-initiated, randomized, multicenter, double-blind, placebo-controlled trial with two treatment arms, starting with 60 mg or 10 mg prednisolone per day, tapered down to 5 mg prednisolone within eight weeks, and one placebo arm, each arm comprising 150 patients. The duration of the intervention is 12 weeks. In parallel, all patients will be treated with methotrexate (usual dosage 15 mg/week). The primary efficacy endpoint is the progression of radiographic joint damage after one year compared to baseline. Important secondary endpoints are the percentage of patients in remission, patient global assessment of disease activity, and changes of functional capacity. Safety monitoring is performed.

The statistical analysis is performed in three hierarchical steps. The first step is an analysis of covariance (α = 0.05) to compare the group with the initial prednisolone dosage of 60 mg and the placebo group. In case of a statistically significant result, the comparison of the group starting with 10 mg prednisolone with the placebo group will be performed as a second step (α = 0.05). In case of superiority of the 10 mg prednisolone group versus the placebo group, the third step will be a non-inferiority test for the 10 mg prednisolone group versus the 60 mg prednisolone group (α = 0.025).

**Discussion:**

The CORRA trial will yield information concerning the optimal glucocorticoid dosage schedule in the treatment of patients with early rheumatoid arthritis.

**Trial registration:**

This trial was registered on 19 November 2013 at ClinicalTrials.gov. Registration number: NCT02000336.

## Background

Rheumatoid arthritis (RA) is a chronic disease characterized by inflammation of the peripheral joints leading to disability, impaired functioning, and premature death. Irreversible joint damage may occur very soon after disease onset. The most important treatment strategy is the early use of disease-modifying antirheumatic drugs (DMARD). However, DMARD have a delayed onset of efficacy and side effects may require cessation of treatment.

Glucocorticoids (GC) are fast-acting anti-inflammatory drugs, now also considered as disease modifying because of their ability to decelerate structural damage [1]. Especially during the first weeks of DMARD treatment, GC are frequently used due to the rapid onset of their anti-inflammatory efficacy. However, it is well known that GC may have considerable side effects such as osteoporosis, diabetes mellitus, arterial hypertension, cardiovascular events, glaucoma, tuberculosis, and weight gain [2]. Hoes *et al*. [3] systematically analyzed the literature on reported adverse events (AE) and found the risk of AE to be 43 per 100 patient-years (95% confidence interval (CI): 30 to 55).

There is good evidence of clinical, functional, and structural efficacy for: 5 to 10 mg prednisolone (PRED)/day over two years [4–7]; and initial dose of 60 mg PRED, tapered to low or zero dose over six to eight months [8, 9] given in addition to DMARD [10]. However, in most rheumatology departments a dosage of 60 mg PRED is not used in the treatment of RA due to toxicity concerns. With the common implementation of ’treat-to-target’ strategies [11], including higher doses of DMARD and a more rapid dose escalation, the percentage of patients undergoing concomitant GC treatment over a long period may diminish. Thus, within a treat-to-target setting, lower dosages of PRED taken over a short period may render sufficient disease modification. Usually, a low (<7.5 mg PRED equivalent a day) to medium (>7.5 mg but <30 mg PRED equivalent) [12]) dose GC schedule is thought to provide sufficient benefit [13]. Following the European League Against Rheumatism (EULAR) recommendations [14], the dose is often tapered as rapidly as is clinically feasible [15].

Therefore, most rheumatologists rather use 7.5 to 10 mg PRED in the initial treatment of RA, increasing the dose in case of lack of efficacy, and tapering the dose over the following weeks to bridge the interval until DMARD become clinically efficacious. Unfortunately, there is insufficient data concerning this dosage schedule.

In order to shed further light on the best possible GC treatment strategy in early RA regarding both efficacy and safety, we have designed a study which may guide future treatment decisions.

The main aims of this study are: to compare the use of GC as a bridging therapy with placebo in a treat-to-target setting with DMARD, and to define the best out of two standard GC dosage regimens. Both aims are with regard to the progression of radiographic joint damage after one year compared to baseline.

## Methods/Design

### Trial design

CORRA is a multicenter, randomized, placebo-controlled, double-blind clinical trial conducted in 30 rheumatology practices in North Rhine-Westphalia, Germany. Most of these practices are members of the RheumaNetz Westfalen-Lippe, a local cooperation of rheumatologists in the eastern part of North Rhine-Westphalia. In total, 450 patients with early active RA will be randomized to receive one of two GC treatments (starting with 10 or 60 mg PRED, tapered down to 5 mg PRED within eight weeks) or placebo, each arm comprising 150 patients. The duration of the intervention is 12 weeks. In parallel, all patients start medication with methotrexate (MTX), at a usual dosage of 15 mg/week. The primary efficacy endpoint is radiographic joint damage after one year compared to baseline. Important secondary endpoints are the percentage of patients in remission, patient global assessment of disease activity, and changes of functional capacity. Safety monitoring is performed.

The study is approved by the local ethic committees Ethik-Kommission der Ärztekammer Westfalen-Lippe und der Medizinischen Fakultät der Westfälischen Wilhelms-Universität Münster (leading ethic committee: 2013-250-f-A), Ethik-Kommission der Ärztekammer Nordrhein, Dusseldorf, and Ethikkommission bei der Ärztekammer Niedersachsen, Hannover. It is registered at the European Union Drug Regulating Authorities (EudraCT: 2012-004074-25), at ClinicalTrials.gov (NCT02000336), and German Clinical Trials Register (DRKS-Nr: DRKS00004774). The principles of good clinical practice will be an essential component of the trial. It will be conducted in accordance with the Declaration of Helsinki. All patients will be required to give written informed consent before inclusion in the study.

### Participants

The 2010 RA classification criteria established by the American College of Rheumatology/EULAR collaborative initiative will be used in this trial to enable the early diagnosis of RA [16]. In general, most patients enrolled in this trial will be seen by a rheumatologist early in the course of their disease. To meet the circumstances of routine rheumatology care where patients are sometimes seen with a delay of many months, patients with a disease duration of up to three years may be enrolled. In general, patients will be MTX naïve (MTX pretreatment of four weeks or less is allowed).

To outweigh the risks of MTX and GC treatment, RA activity must be moderate or high as measured by a Disease Activity Score 28 (DAS28) of >4. The DAS28 score is a compound score comprising the number of tender joints (out of 28), swollen joints (out of 28), the erythrocyte sedimentation rate (ESR, after 1 hour), and the patient’s assessment of disease activity. The range of the score is 0 (no activity) to 10 (maximum disease activity) [17]. In patients with a pain disorder such as fibromyalgia, the DAS28 score may be unduly elevated because of the high number of tender joints. To ensure a sufficient amount of inflammatory disease activity, the number of swollen joints should be three or more for a patient to be enrolled in the trial.

Key exclusion criteria will ensure that no patients with very high risks for GC or MTX toxicity will participate in the trial. The exclusion criteria are in accordance with standard rheumatological decision-making and therefore do not promote undue patient selection for this study.

To avoid an inappropriate risk of MTX toxicity, it is mandatory to exclude patients with severe liver disease, active hepatitis B or C viral infection, renal disease (creatinine clearance <30 ml/minute), clinically relevant hematological disease, relevant immunodeficiency including HIV infection, clinically significant pulmonary fibrosis, complicated gastric or duodenal ulcer, a history of malignant melanoma, pregnancy or planned pregnancy, or severe infections within the previous six weeks before study participation.

Patients with uncontrolled diabetes mellitus, arterial hypertension, or intraocular pressure should not receive GC treatment and are therefore excluded as well. On general grounds, non-compliant patients and patients outside the range of 18 to 80 years will not be allowed to participate.

### Study medication, randomization, and follow-up

The intervention phase will last 84 days. The respective PRED dosages of the treatment groups are listed in Table 1.

**Table 1:** **Schedule of the prednisolone dosage (mg/day) of the three study arms of the CORRA study**

Day	Placebo	V10	V60
1 - 7	0	10	60
8 - 14	0	10	40
15 - 21	0	10	25
22 - 28	0	10	20
29 - 35	0	7,5	15
36 - 42	0	7,5	10
43 - 49	0	7,5	7,5
50 - 56	0	7,5	7,5
57 - 84	0	5	5

CORRA: Comparison of the efficacy and safety of two starting dosages of prednisolone in early active rheumatoid arthritis, PRED: prednisolone, V10: group starting with 10 mg PRED; V60: group starting with 60 mg PRED.

In parallel, all patients will start MTX medication at a usual dosage of 15 mg/week (modifications may be made by the rheumatologist, for example due to minor renal insufficiency or age).

Patients are seen every four weeks within the first three months. In case of lack of efficacy, the MTX dose will be increased to 20 mg/week and further up to 25 mg/week, after four or eight weeks, respectively. In the further course of the study, a change of DMARD medication according to the EULAR recommendations [14] is allowed in case of lack of efficacy and/or intolerable side effects. These decisions are made by the treating rheumatologist based on the treat–to-target paradigm for RA [11].

**Figure 1:**
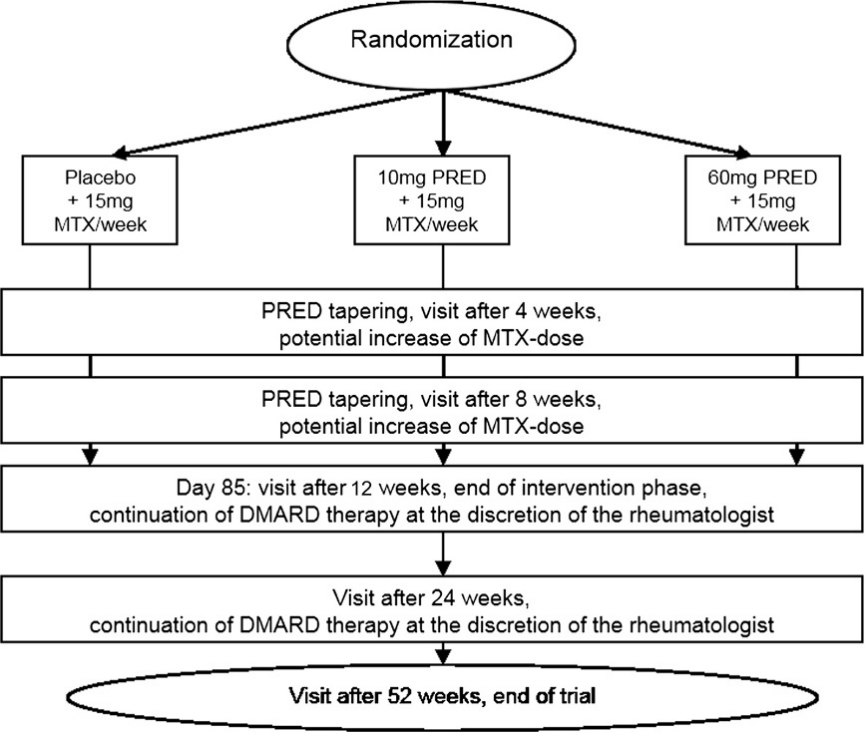
**Schedule of the CORRA study.** CORRA: Comparison of the efficacy and safety of two starting dosages of prednisolone in early active rheumatoid arthritis, DMARD: disease-modifying antirheumatic drug, MTX: methotrexate, PRED: prednisolone.

Study visits will take place at baseline and after 4, 8, 12, 24, and 52 weeks (Figure 1). Physical examination, determination of DAS28, and safety measures including lab examinations will be performed at every visit. Radiographs of hands and feet will be done at baseline and after one year. Functional assessments including the EuroQol-5D [18], Patient Health Questionnaire-2 [19], and physical functioning questionnaire Hannover (FFbH) [20] are planned at baseline and after 12, 24, and 52 weeks. Dual-Energy X-ray Absorptiometry (DXA) of the lumbar spine and hip at baseline and after 24 weeks is planned in case of at least one of the following: age >60 years for men, >50 years for women, current smoker, history of non-traumatic fracture, parental history of hip fracture (Table 2).

**Table 2:** **Scope of study visits and assessments of the CORRA study**

	Physical examination, DAS28, laboratory, safety assessment	Radiographs of hands and feet	Patient global assessment, functional capacity, EQ-5D	DXA lumbar spine and hip
Baseline	+	+	+	(+)
Week 5	+			
Week 9	+			
Week 13	+		+	
Week 25	+		+	(+)
Week 53	+	+	+	

CORRA: Comparison of the efficacy and safety of two starting dosages of prednisolone in early active rheumatoid arthritis, DAS28: Disease Activity Score 28, EQ-5D: EuroQol-5D, DXA: Dual-Energy X-ray Absorptiometry.

Allocation concealment is ensured as the randomization code will not be released until the end of the study. Randomization will be performed using blocks with variable block length according to centre. Quality assurance and monitoring will be performed in 100% of data for the inclusion and exclusion criteria, verification of treatment compliance, primary endpoint, and AE. Other trial data will undergo 100% source data verification for 20% of patients.

The treating rheumatologist will record all AE including serious AE and, if necessary, make treatment adjustments in accordance with the protocol. Serious AE are defined as any adverse reaction resulting in any of the following outcomes: a life-threatening condition or death, a significant or permanent disability, a malignancy, hospitalization or prolongation of hospitalization, a congenital abnormality, or a birth defect.

Supervision of the trial will be performed by an independent data and safety monitoring board (DSMB). In case of unexpected safety concerns the biostatistician of the DSMB will perform an interim analysis. The committee will ensure adherence to protocol and provide the funding organization with information and advice.

### Primary outcome parameter

One of the best evaluated and relevant outcome measures in the assessment of RA is the radiographic joint damage as measured by the van der Heijde modification of the Sharp score (SHS) [21]. This score reflects the structural damage caused by the inflammatory joint disease.

Radiographic joint damage, as assessed by the SHS or other scoring systems, correlates with physical function [22], employment status [23], and mortality [24]. The effects discussed in the literature are comparable to the effects assumed for this study. According to the ‘window of opportunity’ concept, reduced radiological damage early in the course of RA leads to a beneficial modification of the long-term outcome. Therefore, the primary outcome parameter will be the progression of radiographic joint damage after one year compared to baseline measured by the SHS.

### Secondary outcome parameter

Secondary endpoints will be: percentage of patients in remission (as defined by DAS28 < 2.6) or in a state of low disease activity (DAS28 ≥ 2.6 and DAS28 < 3.2) both at week 13 and after one year, changes of FFbH at week 13 and after one year, decline in bone mineral density (DXA lumbar spine and hip) at six months compared to baseline, number and overall dose of GC injections, direct costs of treatment over one year, mean MTX dosage, patient global assessment of disease activity, and percentage of patients who switch to biological treatment in cases of inefficacy of conventional DMARD therapy.

Among all secondary endpoints, the DAS28 is the most important since it is a well-established tool used to assess disease activity in RA. In daily routine, it is frequently used to guide therapeutic decision-making. Similarly, the FFbH is a validated tool to measure physical functioning which is considered to be one of the most important prognostic markers in RA [20]. To assess the risk of osteoporotic fractures, the measurement of bone mineral density (done by DXA) is recommended by the German ‘Dachverband Osteologie’. To estimate the economic burden of RA, direct costs of treatment per month over one year will be determined. To take into account the total intra-articular dose of GC, the number and overall dose of GC injections is recorded. As a lower GC dosage may lead to an increase in MTX dosage, the mean MTX dosage will be a secondary endpoint. To provide patients’ perspective of the effects of the different treatment arms, patient global assessment of disease activity is selected as a secondary endpoint. As treatment with biologicals leads to reduced radiographic joint damage that is comparable to the disease modifying effect of GC, the percentage of patients who switch to a biological treatment in cases of inefficacy of conventional DMARD therapy will be assessed as a secondary endpoint. Finally, a secondary analysis of the primary outcome parameter will be performed, adjusting for mean MTX dose, use of biologicals, and mean GC dose during weeks 13 to 52.

### Sample size

It is assumed that progression of radiographic joint damage as measured by the SHS will be the same for both dosage levels of PRED. For sample size calculation we used data from the BeSt-study [9]. We thus assume an effect of 3.7 and a standard deviation of 7.5. Accounting for a 5% dropout rate, the effect will be 3.5. If 150 patients per group are included, a two sided t-test of superiority (α = 0.05) between one of the PRED groups and the placebo group will give a power of >0.95. Accepting a non-inferiority margin of 1.3 (one third of the known placebo-verum difference) for the difference in progression between the PRED groups, the one sided non-inferiority test in step three (at α = 0.025) will give a power of >0.80, if 149 patients per PRED group are included. The significance level of α = 0.025 was chosen according to the European Medicines Agency guidelines [25]. Thus, the final sample size has been set as 150 patients per group.

### Blinding and statistical methods

All participating rheumatologists and radiologists will be blinded against the interventions, thus assuring blinded assessment of outcome variables.

**Figure 2:**
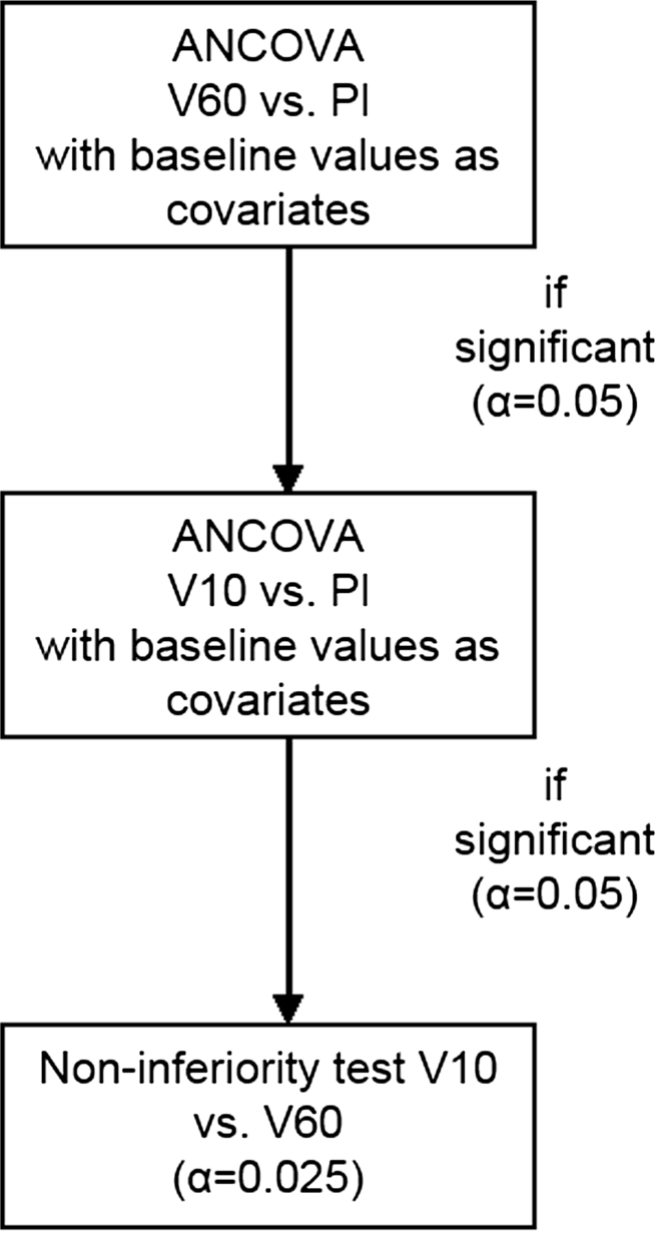
**Statistical analyses procedure of the CORRA study.** CORRA: Comparison of the efficacy and safety of two starting dosages of prednisolone in early active rheumatoid arthritis, Pl: placebo group, V10: group starting with 10 mg prednisolone daily, V60: group starting with 60 mg prednisolone daily.

The primary endpoint is the progression of radiographic damage after one year compared to baseline (measured by the SHS). Analysis will be performed in three steps to compare the three groups (Pl: placebo group; V10: group starting with 10 mg PRED; and V60: group starting with 60 mg PRED). Primary analysis will be conducted by analysis of covariance (ANCOVA) for V60 versus Pl, using baseline values as covariates and 12-month values as dependent variables. In case of a statistically significant result (α = 0.05), a comparison of V10 versus Pl will be performed (α = 0.05). In case of superiority of V10 versus Pl, a non-inferiority test for V10 versus V60 will be performed (α = 0.025), as is shown in Figure 2. A non-inferiority margin of 1.3 SHS points has been selected.

Statistical analysis will be performed using an intention-to-treat (ITT) approach. It is expected that 12-month results will be missing for less than 5% of the patients (dropout rate). In step one and two, missing radiographs after one year are substituted by randomly selected radiographs of patients of the placebo group (both replacements by the same patient), thus leading to conservative results for the PRED groups. In step three of the test procedure, missing values will be excluded from the analysis. A sensitivity analysis will be done for the effect of the replacement procedure (the random replacement will be repeated several times).

Analysis of the secondary outcome parameters will be performed by ITT using all available data. Continuous characteristics of the groups will be compared using analysis of covariance adjusting for baseline data. Analysis of categorical variables or proportions will be performed using the chi-square test. An additional repeated measures analysis will be done for all quantitative endpoints taken at several time points.

## Discussion

The most frequently used dosage of 7.5 to 10 mg PRED as a bridging treatment in early RA is a compromise between the good clinical short-term effect of GC and the attempt to minimize the risk of AE. However, the first months of RA are considered to be like a window of opportunity, with the best offer to change the course of the disease. This window of opportunity should be used to ‘hit hard and early’ (provide aggressive treatment as soon as possible) [14]. It may well be that higher dosages of GC, as used in the COBRA study [8] and the BeSt-study [9], have a higher impact on the outcome without a considerable increase of AE. The answer to this question would be highly relevant for the individual patient because of the increased possibility to induce a more favorable course of the disease. The immediate benefits for society in general and for the economic expenditures in particular are obvious.

The aim of our study is to test different treatment strategies rather than to evaluate the dose-dependent effect of PRED on the Sharp score. This means to find answers to two questions. Firstly, is it worthwhile to start with a high-dose PRED treatment in active RA patients, even if a treat-to-target approach is followed? Secondly, is there any difference between initially high and low PRED doses in the sense of benefit in radiographic progression? In the further course, there are many potential confounders, such as imbalanced MTX use, imbalanced GC use during weeks 13 to 52, and imbalanced use of biologics. These imbalances may flaw the immediate effect of the PRED bridging on the Sharp score, but have only a minor impact on the tested treatment strategies. The possible results of our testing procedure may clarify if high-dose PRED bridging is not superior to a placebo, then the disease modifying effect of PRED no longer plays a major role in a treat-to-target setting. If there is a significant difference between high-dose PRED bridging and placebo, and low-dose PRED bridging is not superior to a placebo, then high-dose bridging may be strongly recommendable. If a low-dose bridging compares to a significantly effective high-dose bridging, the EULAR recommendation of merely regarding the symptomatic effect of PRED is confirmed. If there are no differences between all three groups even with sufficiently imbalanced concomitant therapies, then only symptomatic effects of PRED should guide treatment decisions.

Of course, these results must be judged by considering other endpoints such as adverse events, clinical response to treatment, and patient-related outcomes. Therefore recommendations should take into account all these results. It is possible, depending on the results, that there may remain sufficient uncertainty and, therefore, that a confirmatory trial may be needed. Nonetheless, the primary endpoint should not be blurred by these short-term results because the focus lies on disease modification as measured by the Sharp score.

## Trial status

Recruitment started in February 2014 and is expected to finish in 2016.

## Abbreviations

AE: Adverse events
CI: Confidence interval
DAS28: Disease Activity Score 28
DMARD: Disease-modifying antirheumatic drug
DRKS: German Clinical Trials Register
DSMB: Data and safety monitoring board
DXA: Dual-Energy X-ray Absorptiometry
ESR: Erythrocyte sedimentation rate
EudraCT: European Union Drug Regulating Authorities
EULAR: European League Against Rheumatism
FFbH: Physical functioning questionnaire Hannover
GC: glucocorticoid
ITT: Intention-to-treat
MTX: methotrexate
Pl: Placebo group
PRED: prednisolone
RA: Rheumatoid arthritis
sd: Standard deviation
SHS: van der Heijde modification of the Sharp score
V10: Group starting with 10 mg prednisolone
V60: Group starting with 60 mg prednisolone.
